# Mechanical properties and biocompatibility of a novel miniscrew made of Zr_70_Ni_16_Cu_6_Al_8_ bulk metallic glass for orthodontic anchorage

**DOI:** 10.1038/s41598-023-30102-3

**Published:** 2023-02-21

**Authors:** Shutaro Sasaki, Masahiro Seiryu, Hiroto Ida, Shunro Miyashita, Nobuo Takeshita, Daiki Irie, Yoshihiko Yokoyama, Teruko Takano-Yamamoto

**Affiliations:** 1 Division of Orthodontics and Dentofacial Orthopedics, Tohoku University Graduate School of Dentistry, 4-1, Seiryomachi, Aoba-ku, Sendai, Miyagi 980-8575 Japan; 2 Section of Orthodontics and Dentofacial Orthopedics, Division of Oral Health, Growth and Development, Faculty of Dental Science, Kyushu University, Fukuoka, Fukuoka Japan; 3 Cooperative Research and Development Center for Advanced Materials, Institute for Materials Research, Tohoku University, Sendai, Miyagi Japan; 4 Department of Biomaterials and Bioengineering, Faculty of Dental Medicine, Hokkaido University, Sapporo, Hokkaido Japan

**Keywords:** Biomedical materials, Implants

## Abstract

The purpose of the present study was to fabricate a miniscrew possible for clinical application using Zr_70_Ni_16_Cu_6_Al_8_ bulk metallic glass (BMG), which has high mechanical strength, low elastic modulus, and high biocompatibility. First, the elastic moduli of Zr-based metallic glass rods made of Zr_55_Ni_5_Cu_30_Al_10_, Zr_60_Ni_10_Cu_20_Al_10_, Zr_65_Ni_10_Cu_17.5_Al_7.5_, Zr_68_Ni_12_Cu_12_Al_8_, and Zr_70_Ni_16_Cu_6_Al_8_ were measured. Zr_70_Ni_16_Cu_6_Al_8_ had the lowest elastic modulus among them. Then, we fabricated Zr_70_Ni_16_Cu_6_Al_8_ BMG miniscrews with diameters from 0.9 to 1.3 mm, conducted a torsion test, and implanted them into the alveolar bone of beagle dogs to compare insertion torque, removal torque, Periotest, new bone formation around the miniscrew, and failure rate compared with 1.3 mm diameter Ti-6Al-4 V miniscrew. The Zr_70_Ni_16_Cu_6_Al_8_ BMG miniscrew exhibited a high torsion torque even if the miniscrew had a small diameter. Zr_70_Ni_16_Cu_6_Al_8_ BMG miniscrews with a diameter of 1.1 mm or less had higher stability and lower failure rate than 1.3 mm diameter Ti-6Al-4 V miniscrews. Furthermore, the smaller diameter Zr_70_Ni_16_Cu_6_Al_8_ BMG miniscrew was shown, for the first time, to have a higher success rate and to form more new bone around the miniscrew. These findings suggested the usefulness of our novel small miniscrew made of Zr_70_Ni_16_Cu_6_Al_8_ BMG for orthodontic anchorage.

## Introduction

Metallic glass, which is an amorphous alloy, has excellent physical, mechanical, and chemical properties^1,2^. In particular, Zr-based metallic glass has been reported to be a material with high strength, low elastic modulus, and high biocompatibility due to high corrosion resistance^3–5^. Yokoyama et al.^6^ have developed Zr_70_Ni_16_Cu_6_Al_8_bulk metallic glass (BMG) with a high Poisson's ratio and low modulus of elasticity. The elastic modulus of Zr_70_Ni_16_Cu_6_Al_8_ BMG (70 GPa)^7^ is smaller than that of Ti-6Al-4 V alloy (100–130 GPa)^8–12^ and is closer to that of cortical bone (12—17 GPa)^13^ than Ti-6Al-4 V alloy (100–130 GPa)^14^. It has also been reported that Zr_70_Ni_16_Cu_6_Al_8_ BMG, which had a high zirconium content, has higher compressive strength than conventional metallic glass^4^.

We have recently analyzed Zr_70_Ni_16_Cu_6_Al_8_ BMG by tensile strength test, corrosion test, ion release test, and cell adhesion, proliferation and differentiation tests, which indicated that Zr_70_Ni_16_Cu_6_Al_8_ BMG has excellent mechanical properties with high strength, low elastic modulus, high corrosion resistance, and bio-affinity^15^. Furthermore, we fabricated a prototype 1.3 mm diameter miniscrew made of Zr_70_Ni_16_Cu_6_Al_8_ BMG and pure titanium, and implanted them in the rat tibia. The results demonstrated that the Zr_70_Ni_16_Cu_6_Al_8_ BMG prototype miniscrew had a significantly lower mobility and a higher removal torque value than pure titanium miniscrews; it also had more osseointegration and new bone formation around the Zr_70_Ni_16_Cu_6_Al_8_ BMG miniscrew^15^.

Recently, pure titanium and titanium alloys have been widely used as metallic biomaterials in clinical practice, and Ti-6Al-4 V alloys are known as the main composition^16^. However, it has been reported that implants made of titanium and titanium alloys have problems such as loosening of the joint with bone in artificial joints^17^ due to stress shielding at the joint between the implant and the bone^18^. It has also been suggested that the reason for the loosening is the difference in elastic modulus with the bone^19^ although the bone itself is viscoelastic^20^.

There are also some problems with the conventional miniscrews for orthodontic anchorage, such as the risk of damage to the adjacent tooth root during implantation^21–23^, low strength such as miniscrew breakage^24,25^, miniscrew mobility and failure during treatment^26,27^, and stability only with mature bone^28^. Thus, the mechanical properties of the Ti-6Al-4 V alloy, such as tensile strength and elastic modulus, and new bone formation around the implanted miniscrew are not always sufficient for clinical use.

We have previously reported that the proximity of the root and the miniscrew is related to the miniscrew failure rate^27,29^. To avoid contact and proximity of the miniscrew to the root, we could hypothesize that it would be effective to reduce the diameter of the miniscrew implanted into a narrow area of alveolar bone between tooth roots. If it is possible to manufacture a miniscrew made of Zr_70_Ni_16_Cu_6_Al_8_ BMG with a smaller diameter and stronger osseointegration than the conventional miniscrew made of titanium alloy, various problems could be improved by higher strength, lower elastic modulus, and possible avoidance of proximity to the tooth root. In particular, stress shielding is less likely to occur between a Zr_70_Ni_16_Cu_6_Al_8_ BMG miniscrew and the surrounding new bone, and then strong osseointegration could be obtained. The aim of the present study was to investigate the potential clinical application of orthodontic miniscrew with the smaller diameter than the currently used Ti and Ti-alloy miniscrews, using Zr_70_Ni_16_Cu_6_Al_8_ bulk metallic glass (BMG), which has high mechanical strength and low elastic modulus, and high biocompatibility.

## Results

### Measurement of elastic modulus and torsion test

**Figure 1:**
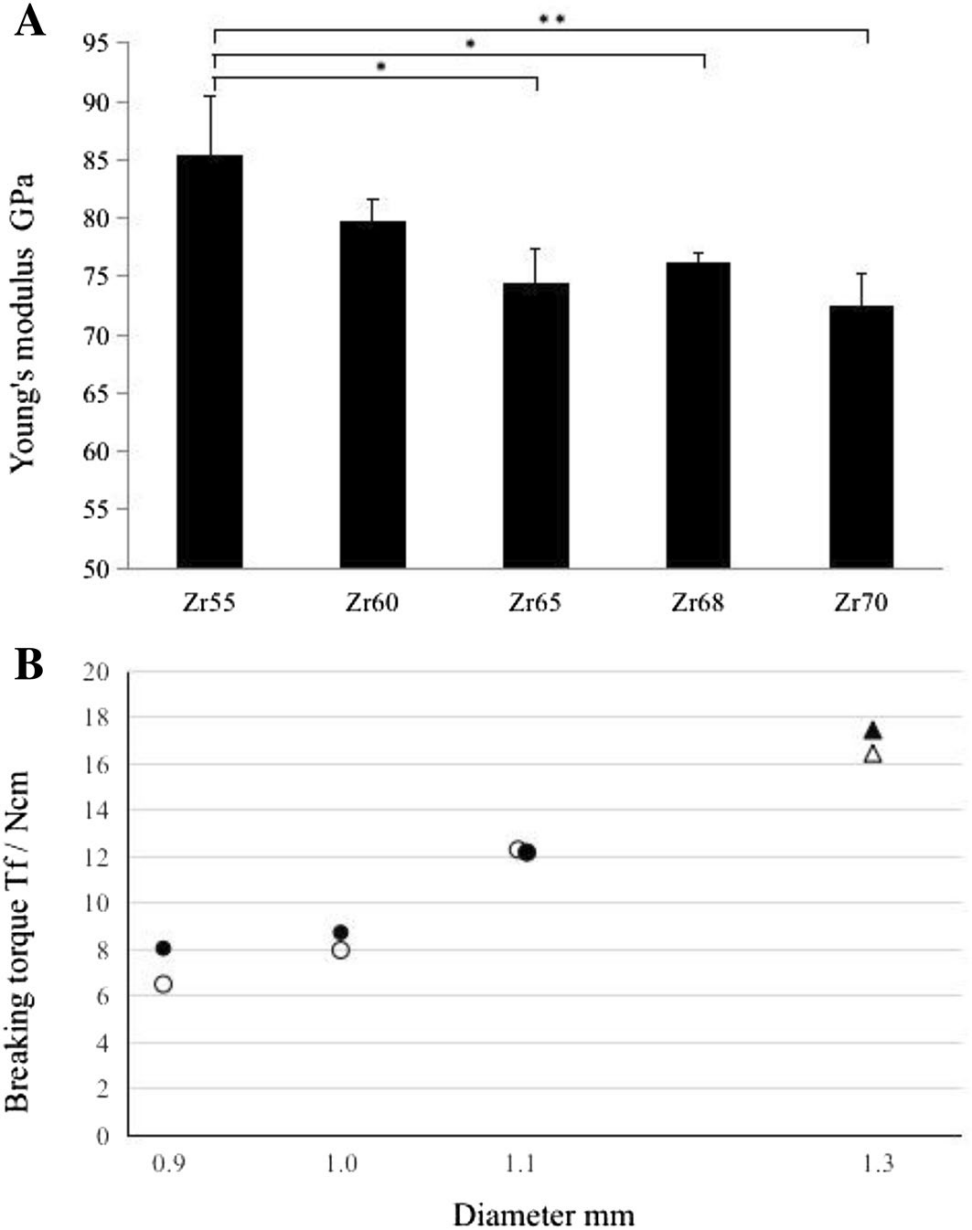
Young’s modules and torsion breaking torque test by a free resonance vibration method. **A** The measurement of Young’s modules by the free resonance vibration method of five types of Zr-based metallic glass rods (Zr_55_Ni_5_Cu_30_Al_10_, Zr_60_Ni_10_Cu_20_Al_10_, Zr_65_Ni_10_Cu_17.5_Al_7.5_, Zr_68_Ni_12_Cu_12_Al_8_, and Zr_70_Ni_16_Cu_6_Al_8_). (n = 3 per group). ^**^P < 0.01, ^*^P < 0.05. (**B**) The torsion breaking torque test of Zr_70_Ni_16_Cu_6_Al_8_ BMG and Ti-6Al-4 V miniscrews unused and used for 8 weeks after implantation. △:Ti-6Al-4 V unused miniscrew, ○:Zr_70_Ni_16_Cu_6_Al_8_ BMG unused miniscrew, ▲:Ti-6Al-4 V miniscrew used for 8 weeks after implantation, ●: Zr_70_Ni_16_Cu_6_Al_8_ BMG miniscrew used for 8 weeks after implantation (n = 3–5 per group).

Measured by a free resonance vibration method, the elastic moduli of Zr_55_Ni_5_Cu_30_Al_10_, Zr_60_Ni_10_Cu_20_Al_10_, Zr_65_Ni_10_Cu_17.5_Al_7.5_, Zr_68_Ni_12_Cu_12_Al_8_, and Zr_70_Ni_16_Cu_6_Al_8_ BMG were 85.43 GPa, 79.8 GPa, 74.5 GPa, 76.1 GPa and 72.5 GPa, respectively (Fig. 1A). Zr_65_Ni_10_Cu_17.5_Al_7.5_, Zr_68_Ni_12_Cu_12_Al_8_, and Zr_70_Ni_16_Cu_6_Al_8_ BMG showed significantly lower elastic moduli than Zr_55_Ni_5_Cu_30_Al_10_ BMG (Fig. 1A). In braking torsion test on unused miniscrews, 0.9 mm, and 1.0 mm diameter miniscrews made of Zr_70_Ni_16_Cu_6_Al_8_ BMG and the 1.3 mm diameter Ti-6Al-4 V miniscrew showed 6.5 Ncm, and 8.0 Ncm, and 16.5 Ncm, respectively (Fig. 1B). In torsion test on used miniscrew, 0.9 mm, 1.0 mm and 1.1 mm diameter miniscrews made of Zr_70_Ni_16_Cu_6_Al_8_ BMG, and the 1.3 mm diameter Ti-6Al-4Vminiscrew were 8.1 Ncm, 8.7 Ncm, 12.1 Ncm and 17.5 Ncm, respectively (Fig. 1B). The torsion torque values of all of the used Zr_70_Ni_16_Cu_6_Al_8_ BMG miniscrews and the used Ti-6Al-4 V miniscrews were similar to the value of the unused miniscrews (Fig. 1B).

### Surface condition of miniscrews observed by scanning electron microscope (SEM)

**Figure 2:**
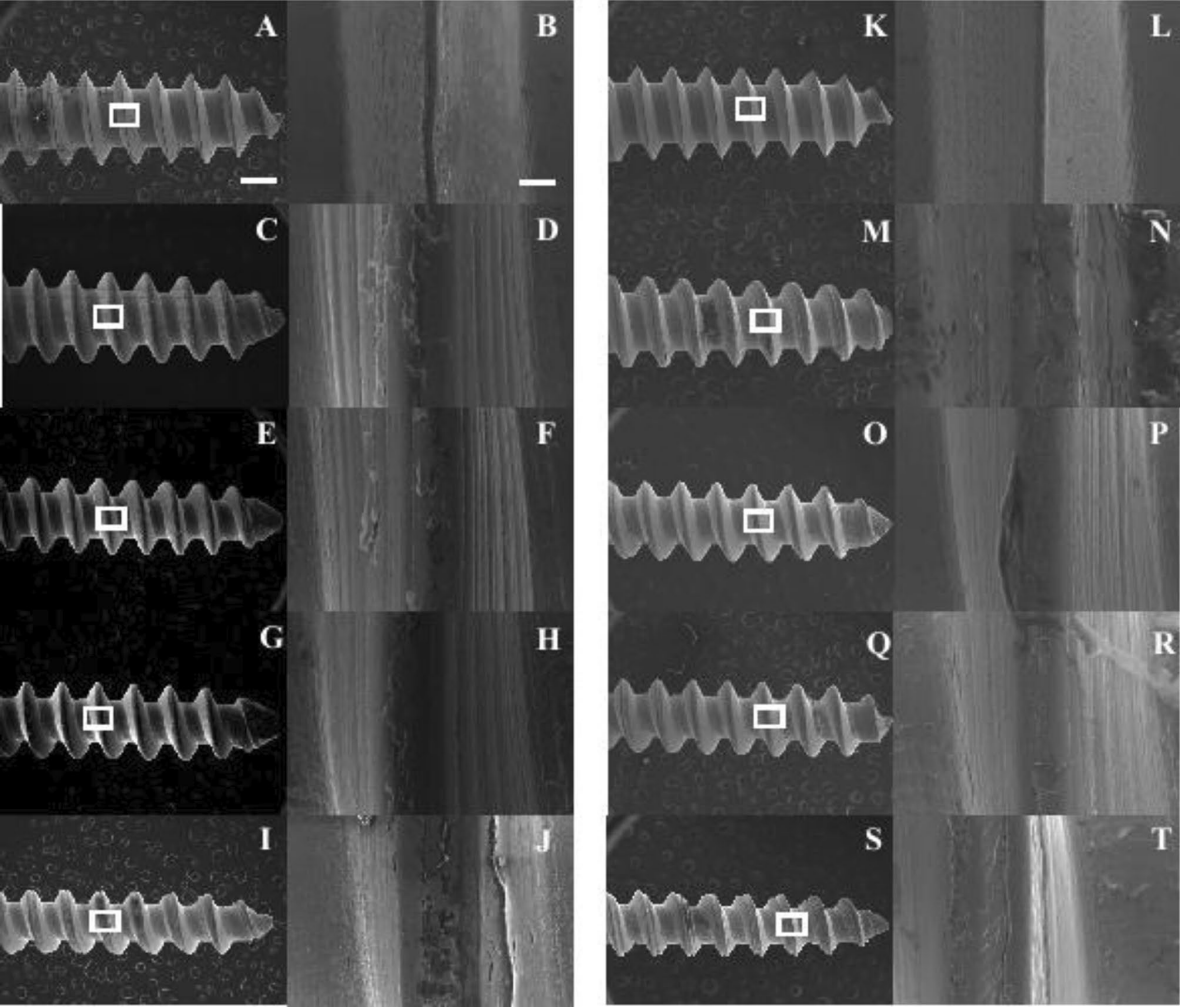
SEM images of unused and used miniscrews for 8 weeks after implantation. (**A**), (**B**), (**C**), (**D**), (**E**), (**F**), (**G**), (**H**), (**I**), and (**J**) Unused miniscrews. (**K**), (**L**), (**M**), (**N**), (**O**), (**P**), (**Q**), (**R**), (**S**), and (**T**) Miniscrews used for 8 weeks after implantation. (**A**), (**B**), (**K**) and (**L**): Ti-6Al-4 V miniscrews. (**C**), (**D**), (**M**) and (**N**): 1.3 mm diameter Zr_70_Ni_16_Cu_6_Al_8_ BMG miniscrews. (**E**), (**F**), (**O**), and (**P**): 1.1 mm diameter Zr_70_Ni_16_Cu_6_Al_8_ BMG miniscrews. (**G**), (**H**), (**Q**), and (**R**): 1.0 mm diameter Zr_70_Ni_16_Cu_6_Al_8_ BMG miniscrews. (**I**), (**J**), (**S**), and (**T**): 0.9 mm diameter Zr_70_Ni_16_Cu_6_Al_8_ BMG miniscrews. White boxes of (**A**), (**C**), (**E**), (**G**), (**I**), (**K**), (**M**), (**O**), (**Q**), and (**S**) were magnified to (**B**), (**D**), (**F**), (**H**), (**J**), (**L**), (**N**), (**P**), (**R**), and (**T**), respectively. Scale bars, (**A**), (**C**), (**E**), (**G**), (**I**), (**K**), (**M**), (**O**), and (**Q**) 500 μm, (**B**), (**D**), (**F**), (**H**), (**J**), (**L**), (**N**), (**P**), (**R**), and (**T**) 50 μm.

SEM images did not show a clear difference in the shape and surface of the miniscrew between the Zr_70_Ni_16_Cu_6_Al_8_ BMG miniscrew and the Ti-6Al-4 V miniscrew (Fig. 2A–J). At 8 weeks post-implantation no major damage was observed on used miniscrews. No obvious damage or deformation was observed on any edge of used miniscrews at high magnification (Fig. 2K–T).

### Insertion and removal torque testing

**Figure 3:**
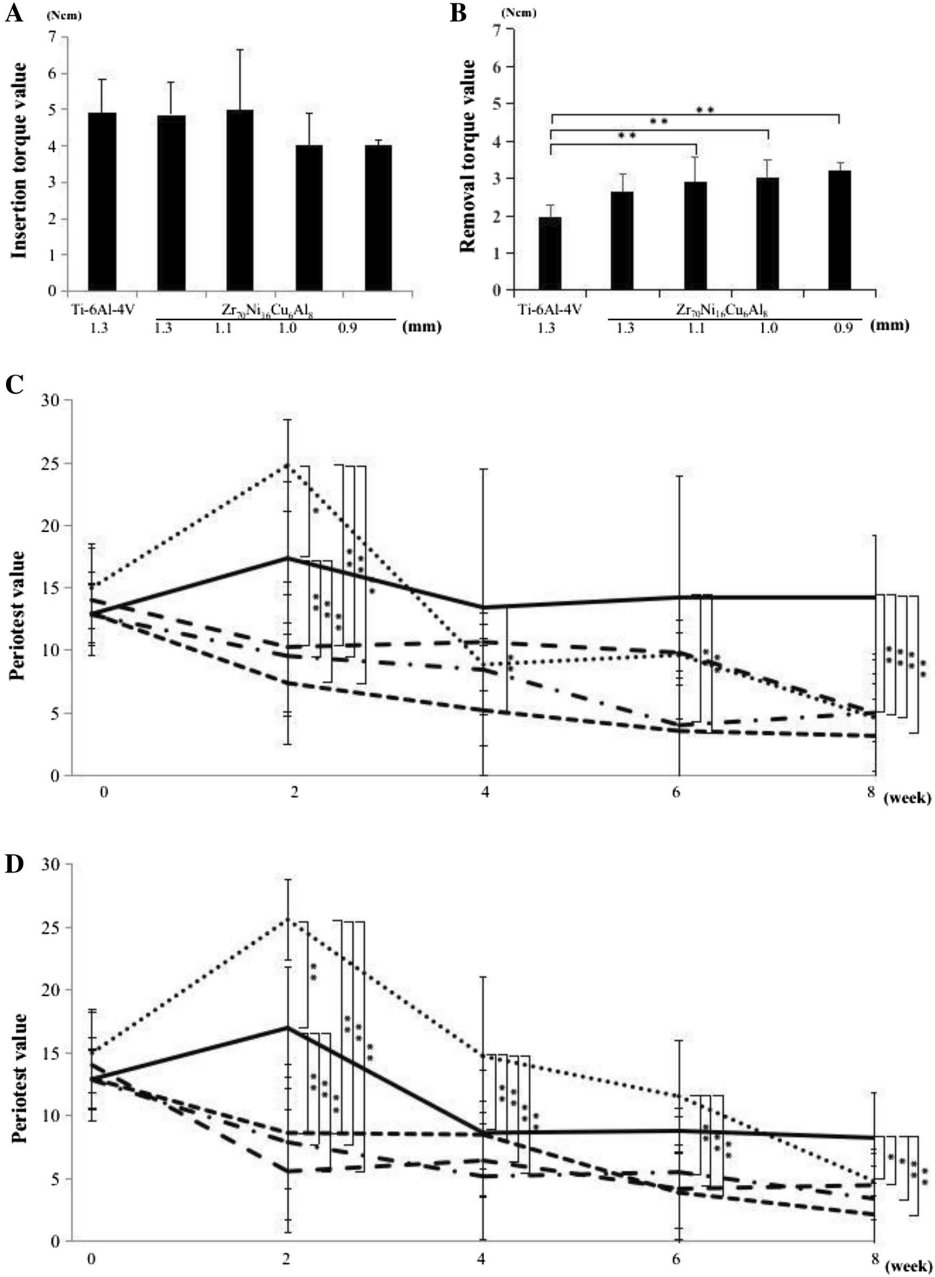
Insertion torque, removal torque and Periotest. (**A**) The insertion torque values of 0.9 mm (n = 20), 1.0 mm (n = 19), 1.1 mm (n = 19), and 1.3 mm (n = 16) diameter Zr_70_Ni_16_Cu_6_Al_8_ BMG miniscrews and 1.3 mm diameter Ti-6Al-4 V miniscrew. (**B**) The removal torque values of 0.9 mm, 1.0 mm, 1.1 mm and 1.3 mm diameter Zr_70_Ni_16_Cu_6_Al_8_ BMG miniscrews and 1.3 mm diameter Ti-6Al-4 V miniscrew, (n = 6 per group) ^**^P < 0.01, ^*^P < 0.05. (**C**) Periotest values of 0.9 mm () (n = 10), 1.0 mm () (n = 9), 1.1 mm () (n = 11), and 1.3 mm () (n = 8) diameter Zr_70_Ni_16_Cu_6_Al_8_ BMG miniscrews and 1.3 mm () diameter Ti-6Al-4 V miniscrew (n = 8) in the non-loaded group. ^**^P < 0.01, ^*^P < 0.05. **D** Periotest values of 0.9 mm () (n = 10), 1.0 mm () (n = 10), 1.1 mm () (n = 8), and 1.3 mm () (n = 8) diameter Zr_70_Ni_16_Cu_6_Al_8_ BMG miniscrews and 1.3 mm () diameter Ti-6Al-4 V miniscrew (n = 8) in the 200 gf loaded group. ^**^P < 0.01, ^*^P < 0.05.

Zr_70_Ni_16_Cu_6_Al_8_ BMG miniscrews with diameters of 0.9 mm and 1.0 mm tended to have a slightly lower insertion torque than miniscrews with diameters of 1.1 mm and 1.3 mm, but no significant difference was observed (Fig. 3A).

In the 200 gf loaded group, the removal torque values of 0.9 mm and 1.0 mm diameter Zr_70_Ni_16_Cu_6_Al_8_ BMG miniscrews were significantly higher than that of the 1.3 mm diameter Ti-6Al-4 V miniscrew (Fig. 3B). A similar trend was seen in the non-loaded group, but there was no significant difference. No significant difference in the removal torque values was observed between the non-loaded and 200 gf loaded groups.

### Stability of Zr_70_Ni_16_Cu_6_Al_8_ BMG miniscrews evaluated by Periotest

Periotest values were measured immediately after implantation, and at 2, 4, 6, and 8 weeks post-implantation to evaluate changes in miniscrew stability. The Periotest values of the 1.3 mm diameter Ti-6Al-4 V miniscrew and the 1.3 mm diameter Zr_70_Ni_16_Cu_6_Al_8_ BMG miniscrew increased significantly from 0 to 2 weeks post-implantation in non-loaded and loaded groups (Fig. 3C,D). Periotest values of the 1.3 mm Ti-6Al-4 V miniscrew did not change from 4 to 8 weeks post-implantation, but the 1.3 mm diameter Zr_70_Ni_16_Cu_6_Al_8_ BMG miniscrew decreased from 2 to 8 weeks post-implantation. The Periotest values of 0.9 mm, 1.0 mm, and 1.1 mm diameter Zr_70_Ni_16_Cu_6_Al_8_ BMG miniscrews decreased significantly with time from 0 to 8 weeks post-implantation, indicating that the increased stability of miniscrews less than 1.1 mm in diameter occurred in a time-dependent manner (Fig. 3C,D). At 8 weeks post-implantation, all Zr_70_Ni_16_Cu_6_Al_8_ BMG miniscrews showed significantly lower Periotest values than the Ti-6Al-4 V miniscrew (Fig. 3C,D). There was no significant difference between the non-loaded and 200 gf loaded groups from 0 to 8 weeks post-implantation (Fig. 3 C,D).

### Histomorphometric analysis around miniscrew

**Figure 4:**
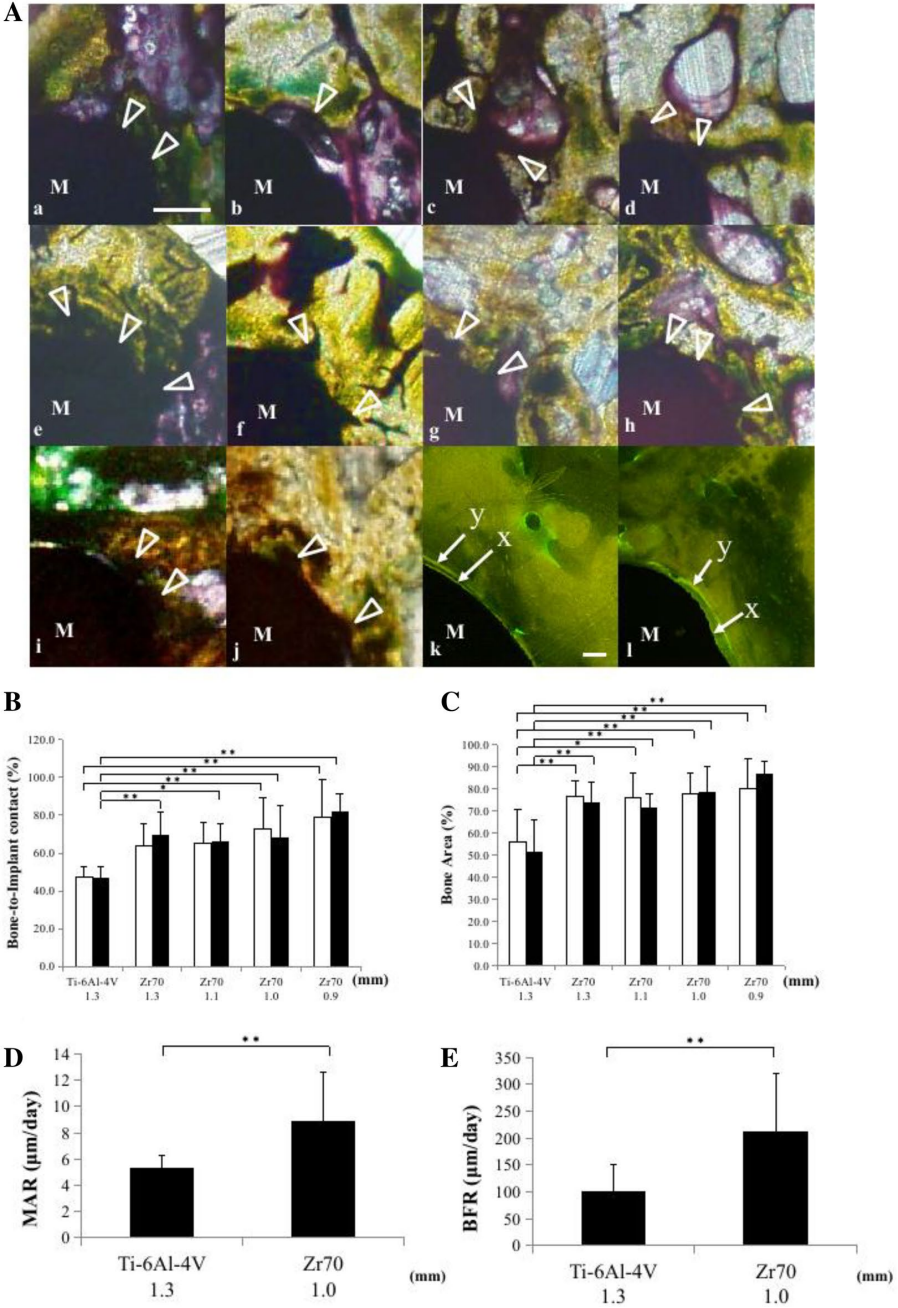
Non-decalcified tissue section image and histomorphometric analysis of bone around the miniscrew. (**A**) The tissue images of 1.3 mm diameter Ti-6Al-4 V miniscrew (**a**, **b**), and 1.3 mm diameter (**c**, **d**), 1.1 mm diameter (**e**, **f**), 1.0 mm diameter (**g**, **h**) and 0.9 mm (**i**, **j**) diameter Zr_70_Ni_16_Cu_6_Al_8_ BMG miniscrews at 8 weeks post-implantation. The tissue images of the non-loaded group were (**a**), (**c**), (**e**), (**g**), and (**i**). The tissue images of the 200 gf loaded group were (**b**), (**d**), (**f**), (**h**), and (**j**). The arrowhead indicates the newly formed bone (dark purple stained area). The fluorescence microscope images of the Ti-6Al-4 V miniscrew and the Zr_70_Ni_16_Cu_6_Al_8_ BMG miniscrew are shown in (**k**) and (**l**), respectively. Calcein green label (x) and tetracycline label (y) are shown. Scale bars, 250 μm: a, b, c, d, e, f, g, h, i, and j, 50 μm: k and l. (**B**): BIC of each miniscrew in the non-loaded group (□) (n = 5 for 1.3 mm, n = 8 for 1.1 mm, n = 6 for 1.0 mm, n = 7 for 0.9 mm diameter) and the 200 gf loaded group (■) (n = 5 for 1.3 mm, n = 5 for 1.1 mm, n = 7 for 1.0 mm, n = 7 for 0.9 mm diameter). ^**^P < 0.01, ^*^P < 0.05. (**C**) BA of each miniscrew in the non-loaded group (□) (n = 5 for 1.3 mm, n = 8 for 1.1 mm, n = 6 for 1.0 mm, n = 7 for 0.9 mm diameter) and the 200 gf loaded group (■) (n = 5 for 1.3 mm, n = 5 for 1.1 mm, n = 7 for 1.0 mm, n = 7 for 0.9 mm diameter). ^**^P < 0.01, ^*^P < 0.05. (**D**) MAR of Ti-6Al-4 V miniscrew and Zr_70_Ni_16_Cu_6_Al_8_ BMG miniscrew (n = 6 per group). ^**^P < 0.01, ^*^P < 0.05. E: BFR of Ti-6Al-4 V miniscrew and Zr_70_Ni_16_Cu_6_Al_8_ BMG miniscrew (n = 6 per group). **P < 0.01, *P < 0.05.

Newly formed bone was observed on the surfaces of Ti-6Al-4 V miniscrew and Zr_70_Ni_16_Cu_6_Al_8_ BMG miniscrews at 8 weeks post-implantation (Fig. 4Aa–j Arrowheads). We quantified new bone formation around miniscrew using a histomorphometric analysis.

At 8 weeks post-implantation, values of bone-to-implant contact (BIC, %; bone surface in contact with the miniscrew (μm)/perimeter of the outer circumference of the miniscrew (μm) × 100) and bone area (BA, %; Bone area 240 μm from the miniscrew surface (μm^2^)/Area in 240 μm from the miniscrew surface × 100), of Zr_70_Ni_16_Cu_6_Al_8_ BMG miniscrews with diameters of 0.9 mm, 1.0 mm, 1.1 mm, and 1.3 mm were significantly larger than those of the 1.3 mm diameter Ti-6Al-4 V miniscrew in both the non-loaded and 200 gf loaded groups (Fig. 4B,C). There was no significant difference in the BIC and BA values between non-loaded and 200 gf loaded groups (Fig. 4B,C). The BIC and BA values increased with decreasing the diameter of Zr_70_Ni_16_Cu_6_Al_8_ BMG miniscrew(Fig. 4B,C).

The fluorescent microscope images revealed the deposition of newly formed bone around both 1.0 mm diameter Zr_70_Ni_16_Cu_6_Al_8_ BMG miniscrew and 1.3 mm diameter Ti-6Al-4 V miniscrew by double labeling with calcein (green) and tetracycline (yellow) (Fig. 4A k, l). Fluorescent double labels around Zr_70_Ni_16_Cu_6_Al_8_ BMG miniscrews were more obvious than that of the Ti-6Al-4 V miniscrew (Fig. 4A k, l). The 1.0 mm diameter Zr_70_Ni_16_Cu_6_Al_8_ BMG miniscrew showed significantly higher mineral appositional rate (MAR, μm/day; Distance between double labeled/Days (7 days)) and bone formation rate (BFR, μm/day; MAR × (double label surface + 1/2 single label surface,)/bone surface × 100) than those of the 1.3 mm diameter Ti-6Al-4 V miniscrew (Fig. 4D,E).

### Correlation between failure rate and root proximity or miniscrew diameter and between miniscrew diameter and root proximity

Ninety miniscrews were used to evaluate failure rate, the root proximity, and miniscrew diameter (Table 1).

**Table 1:** Evaluation of miniscrew failures and root proximity.

	**^,^ *Diameter (mm)	Applied force (gf)	Total (n)	*Failure rate (%)	**Proximity (%)	Success (n)		Failure (n)	
						Non-proximity	Proximity	Non-proximity	Proximity
Ti-6Al-4 V	1.3	0	8	50.0	50.0	4	0	0	4
		200	8	50.0		4	0	0	4
Zr_70_Ni_16_Cu_6_Al_8_	1.3	0	8	50.0	56.3	4	0	0	4
		200	8	50.0		3	1	0	4
	1.1	0	11	36.4	42.1	7	0	1	3
		200	8	37.5		2	3	1	2
	1.0	0	9	11.1	21.1	6	2	1	0
		200	10	30.0		7	0	1	2
	0.9	0	10	30.0	36.4	6	1	0	3
		200	10	20.0		7	1	1	1

**P < 0.01 diameter vs failure rate by Pearson’s correlation coefficient test.

*P < 0.05; diameter vs proximity by Pearson’s correlation coefficient test.

In the non-loaded group at 8 weeks post-implantation, the failure rates for Zr_70_Ni_16_Cu_6_Al_8_ BMG miniscrews with diameters of 0.9 mm, 1.0 mm, 1.1 mm, and 1.3 mm and Ti-6Al-4 V miniscrew with diameter of 1.3 mm were 30.0% (3 out of 10 miniscrews), 11.1% (1 out of 9 miniscrews), 36.4% (4 out of 11 miniscrews), 50.0% (4/8 miniscrews), and 50.0% (4 out of 8 miniscrews), respectively (Table 1). In the 200 gf loaded group at 8 weeks post-implantation, the failure rates for Zr_70_Ni_16_Cu_6_Al_8_ BMG miniscrews with diameters of 0.9 mm, 1.0 mm, 1.1 mm, and 1.3 mm and Ti-6Al-4 V miniscrew with diameter of 1.3 mm were 20.0% (2 of 10 miniscrews), 30.0% (3 of 10 miniscrews), 37.5% (3 of 8 miniscrews), 50.0% (4 of 8 miniscrews), and 50.0% (4 of 8 miniscrews), respectively (Table 1). There was a correlation between failure rate and miniscrew diameter by Pearson's correlation coefficient test (*P* = 0.0056, *P* < 0.01), indicating that the miniscrew failure rate decreased with decreasing miniscrew diameter (Table 1).

The root proximity of 0.9 mm, 1.0 mm, 1.1 mm, and 1.3 mm diameter Zr_70_Ni_16_Cu_6_Al_8_ BMG miniscrews and 1.3 mm diameter Ti-6Al-4 V miniscrew was 36.4% (6 out of 20 miniscrews), 21.1% (4 out of 19 miniscrews), 42.1% (8 out of 19 miniscrews), 56.3% (9 out of 16 mini screws), and 50.0% (8 out of 16 miniscrews), respectively (Table 1). There was a correlation between miniscrew diameter and root proximity by Pearson's correlation coefficient test (*P* = 0.025, *P* < 0.05), indicating that root proximity decreased with a decrease in miniscrew diameter (Table 1).

84.4% of failed miniscrew (27 out of 32 miniscrews) showed root proximity, and 86.2% of successful miniscrew (50 out of 58 miniscrews) showed root non-proximity (Table 1). There was a correlation between miniscrew failure rate and root proximity by chi-squared or Fisher’s exact probability test (*P* = 0.00000002, *P* < 0.01), indicating that miniscrew failure rate decreased with decreasing root proximity.

96.9% (31 of 32 miniscrews) of failed miniscrew fell out at 2 weeks (25 miniscrews) and 4 weeks (6 miniscrews) post-implantation (Table 2). There was no correlation between loading or non-loading and miniscrew failure rate by chi-squared or Fisher’s exact probability test (*P* = 0.4245) (Table 2).

**Table 2:** Miniscrew failures during 2–8 weeks after implantation.

	Diameter (mm)	Applied force (gf)	Failure (n)				
			Total	2 weeks	4 weeks	6 weeks	8 weeks
Ti-6Al-4 V	1.3	0	4	4	0	0	0
		200	4	3	1	0	0
Zr_70_Ni_16_Cu_6_Al_8_	1.3	0	4	1	3	0	0
		200	4	2	2	0	0
	1.1	0	4	4	0	0	0
		200	3	3	0	0	0
	1.0	0	1	1	0	0	0
		200	3	2	0	0	1
	0.9	0	3	3	0	0	0
		200	2	2	0	0	0

### Measurement of metal concentration in beagle dogs’ venous blood

To evaluate the potential toxicity of the 1.0 mm diameter Zr_70_Ni_16_Cu_6_Al_8_ BMG miniscrew and the 1.3 mm diameter Ti-6Al-4 V miniscrew in vivo, we measured metal concentrations in beagle dogs’ venous blood by inductively coupled plasma mass spectrometry (ICP-MS) at 8 weeks post-implantation.

The concentration of each metal was at the same level for the control, the Zr_70_Ni_16_Cu_6_Al_8_ BMG miniscrew, and the Ti-6Al-4 V miniscrew at 8 weeks post-implantation (Table 3). There was no significant difference in the metal concentration of titanium, aluminum, vanadium, nickel, copper and zirconium before implantation and 8 weeks after implantation between Zr_70_Ni_16_Cu_6_Al_8_ BMG miniscrew group and the Ti-6Al-4 V miniscrew group (Table 3).

**Table 3:** Measurement of metal concentration in beagle dog blood (μg/g).

	Ti-6Al-4 V		Zr_70_Ni_16_Cu_6_Al_8_	
	0 weeks	8 weeks	0 weeks	8 weeks
Al	3.25 ± 0.13	5.23 ± 2.92	3.62 ± 0.48	3.78 ± 0.40
V	0.03 ± 0.01	0.01 ± 0.02	0.04 ± 0.01	0.06 ± 0.03
Ni	0.52 ± 0.02	0.72 ± 0.66	1.22 ± 0.96	0.61 ± 0.16
Cu	2.94 ± 0.03	3.19 ± 0.62	3.25 ± 0.01	3.95 ± 0.25
Zr	0.05 ± 0.00	0.03 ± 0.01	0.07 ± 0.03	0.04 ± 0.00
Ti	0.11 ± 0.05	0.06 ± 0.02	0.23 ± 0.08	0.11 ± 0.02

Data are expressed as mean ± SD.

## Discussion

The present study was to investigate the potential clinical application of orthodontic miniscrew with the smaller diameter than the currently used Ti and Ti-alloy miniscrews, using Zr_70_Ni_16_Cu_6_Al_8_ bulk metallic glass (BMG), which has high mechanical strength and low elastic modulus, and high biocompatibility. We demonstrated that Zr_70_Ni_16_Cu_6_Al_8_ BMG can be machined into fine screws with a diameter of 1.3 mm or less, having sufficient mechanical strength, higher stability, and lower failure rate compared to the 1.3 mm diameter Ti-6Al-4 V miniscrew which has been widely used in clinical practice. Furthermore, the smaller diameter Zr_70_Ni_16_Cu_6_Al_8_ BMG miniscrew was shown for the first time to form more new bone around the miniscrew and to have a higher success rate.

In the present study, the elastic modulus of Zr-based metallic glass rods with Zr content of 65% or more such as Zr_65_Ni_10_Cu_17.5_Al_7.5_, Zr_68_Ni_12_Cu_12_Al_8_, and Zr_70_Ni_16_Cu_6_Al_8_ was significantly smaller than that of Zr_55_Ni_5_Cu_30_Al_10_ BMG rods. Among these, the elastic modulus of Zr_70_Ni_16_Cu_6_Al_8_ BMG rods was the smallest value, at 72.5 GPa, which was similar to the value reported by Yokoyama et al. (70 GPa)^7^. Zr_70_Ni_16_Cu_6_Al_8_ BMG rods showed a value closer to the elastic modulus of cortical bone (12–17 GPa)^13^ than Ti-6Al-4 V alloy (100–130 GPa)^8–12^ Therefore, we decided to create the prototype miniscrew made of Zr_70_Ni_16_Cu_6_Al_8_ BMG which had the smallest elastic modulus, and evaluate its usefulness as a miniscrew for orthodontic anchorage.

Recently, Ti miniscrew and Ti alloy miniscrews have been widely used in clinical practice, but these miniscrews have been reported to break during implantation and removal^25,30,31^, and thus their strength is not always sufficient. In the present study, the torsion breaking torque value of the 1.1 mm diameter Zr_70_Ni_16_Cu_6_Al_8_ BMG miniscrew was 12.2 Ncm, and those of the 1.0 mm and 0.9 mm diameter miniscrews were about 8.0 Ncm. Those were lower than 15.6 Ncm (155.55 Nmm) for the 1.3–1.4 mm diameter Ti-6Al-4 V miniscrew reported by Wilmes et al^32^. However, the torsion breaking torque value of the 1.1 mm diameter Zr_70_Ni_16_Cu_6_Al_8_ BMG miniscrew performs as well or better than other widely used miniscrews, such as the 1.4 mm diameter Ti-4Al-4 V mini screw (1.23 ± 0.07 kgf•cm, approximately equal to 12.1 Ncm)^33^ and the 1.2 mm diameter pure titanium miniscrew (0.71 ± 0.05 kgf•cm, approximately equal to 7.0 Ncm)^34^. In addition, the torsion breaking torque values for all Zr_70_Ni_16_Cu_6_Al_8_ BMG miniscrews examined had similar values for unused and used. In the SEM image of used miniscrews, no damage or deformation was observed on the surface condition or each edge of the Ti-6Al-4 V miniscrew or Zr_70_Ni_16_Cu_6_Al_8_ BMG miniscrews. These findings indicated that the Zr_70_Ni_16_Cu_6_Al_8_ BMG miniscrew has sufficient strength for clinical use even with a small diameter of 1.1 mm or less.

The initial stability may not be obtained if the implant placement torque is very small, and later stability supported by osseointegration may not be acquired if the implant placement torque is very large^35^. Very tight placement torque can generate a high level stress to result in degeneration of the bone at the implant–tissue interface^36^. In the previous study, we implanted a 1.3 mm diameter Ti-6Al-4 V miniscrew into the human mandible and evaluated the stability of the miniscrew^29^. As a result, good stability was obtained when the insertion torque value was less than 10.0 Ncm, but the failure rate increased when the insertion torque value increased to 10.0 Ncm or more^29^. In the present study, the insertion torque value of each Zr_70_Ni_16_Cu_6_Al_8_ BMG miniscrew was less than 10 Ncm when the predrilling was performed using a round bar of a size suitable for the diameter of each miniscrew. Then, no significant difference was observed between insertion torque values of miniscrews with various diameters.

The removal torque value was used as one of the indexes to evaluate the degree of osseointegration^37^. In general, removal torque value of a miniscrew increased in proportion to its diameter^38^. However, in the present study, the Zr_70_Ni_16_Cu_6_Al_8_ BMG miniscrew with a smaller diameter of 1.0 mm or less showed a significantly larger removal torque value than 1.3 mm diameter Ti-6Al-4 V miniscrew at 8 weeks post-implantation, suggesting that 1.0 mm or less diameter Zr_70_Ni_16_Cu_6_Al_8_ BMG miniscrews form stronger osseointegrations than 1.3 mm diameter Ti-6Al-4 V miniscrew. This suggestion was substantiated by bone histomorphometric analysis. At 8 weeks post-implantation, bone histomorphometric parameters such as BIC, BA, MAR and BFR indicated significantly increased osseointegration and new bone formation around the Zr_70_Ni_16_Cu_6_Al_8_ BMG miniscrews with 1.3 mm or less in diameter than 1.3 mm Ti-6Al-4 V miniscrew. Furthermore, BIC and BA increased inversely with its diameter, indicating that smaller diameter screws have more osseointegration and bone formation around the miniscrew.

Periotest values for dental implants were considered to be insufficient osseointegration if the value was 9 or higher^39^. In the present study, the Zr_70_Ni_16_Cu_6_Al_8_ BMG miniscrew of each diameter showed Periotest values of 5.0 or less in both the non-loaded group and the 200 gf loaded group at 8 weeks post-implantation. The Zr_70_Ni_16_Cu_6_Al_8_ BMG miniscrews showed significantly lower Periotest values and higher stability than the Ti-6Al-4 V miniscrew at 8 weeks post-implantation. This was also consistent with bone histomorphometric analysis at 8 weeks post-implantation. It suggests great potential for clinical application of Zr_70_Ni_16_Cu_6_Al_8_ BMG miniscrew.

Chen et al.^21^ implanted 72 titanium screws in the mandibles of 6 mongrel adult dogs in contact with the roots of adjacent teeth, and observed, histologically, the roots and periodontium over time. In the process, inflammation was observed in the adjacent area of the tooth root that was not directly damaged, and caused bone resorption and bone remodeling. In the present study, it was suggested that the 1.3 mm diameter Ti-6Al-4 V miniscrew and the 1.3 mm Zr_70_Ni_16_Cu_6_Al_8_ BMG miniscrew tended to be close to the adjacent tooth root and then inflammation occurred around there, and that osteoclast activity and bone resorption increased up to 2 weeks post-implantation. These were consistent with increased Periotest values up to 2 weeks post-implantation. On the other hand, it was suggested that miniscrews of 1.1 mm or less were unlikely to be close to the tooth roots and they do not interfere with normal remodeling of the surrounding bone during 2 weeks post-implantation. These are considered to be the mechanisms by which the small-diameter miniscrews are less likely to fall out at 2 weeks post-implantation.

Harmankaya et al.^40^ implanted titanium miniscrews with several coatings and pure titanium miniscrews on the rat tibia and analyzed the gene expression around the miniscrews. As a result, osteoblast markers such as osteocalcin continued to rise until 28 days after implantation, whereas cathepsin K, which was an osteoclast marker, continued to rise until 7 days after implantation, but then declined. In the present study using beagle dogs, Periotest value of the 1.3 mm diameter Ti-6Al-4 V miniscrew and 1.3 mm diameter Zr_70_Ni_16_Cu_6_Al_8_ BMG miniscrew significantly increased at 2 weeks post-implantation in alveolar bone, and most of the screws that fallen out at 2–4 weeks post-implantation. These findings suggested that the initial stability of a 1.3 mm diameter miniscrew was low because osteoclasts were activated due to acute inflammation from bone wound after implantation; however, as the osteoclast activity decreased, osteoblast activity with new bone formation continued to increase until 8 weeks, and then the stability of the miniscrew increased in the late phase of bone wound healing. On the other hand, miniscrews with a diameter of 1.1 mm or less showed no increase in Periotest values, and miniscrew failure was low at 2 weeks post-implantation and zero at 4 weeks post-implantation, suggesting that strong inflammation around the small-miniscrew did not occur to the extent that the screw fell off.

Watanabe et al.^27^ implanted 190 Ti-6Al-4 V miniscrews with a diameter of 1.3 mm in the human mandible and analyzed the relationship between the proximity to the root evaluated by CBCT and the miniscrew failure rate, and suggested that proximity to the root affected miniscrew failure and interfered with the bone remodeling process. In the present study, too, the chi test showed a correlation between the proximity to the tooth root and the miniscrew failure rate. In addition, the 1.3 mm diameter Ti-6Al-4 V miniscrew and the 1.3 mm diameter Zr_70_Ni_16_Cu_6_Al_8_ BMG miniscrew showed a failure rate of 50%, while 1.1 mm, 1.0 mm, and 0.9 mm diameter Zr_70_Ni_16_Cu_6_Al_8_ BMG miniscrews showed 36.8%, 21.1% and 25.0%, respectively. Pearson's correlation coefficient test also showed a correlation between miniscrew diameter and failure rate. These findings indicated that the failure rate of a miniscrew decreased in proportion to its diameter.

Among the constituent elements of the Zr_70_Ni_16_Cu_6_Al_8_ BMG miniscrew, Ni was known to be the most common sensitizer^41–44^. Kinbara et al.^45^ investigated the threshold of Ni allergic concentration in mice and found that sensitization required an Ni concentration of 1,296,000 ppb and induction required an Ni concentration of 1296 ppb. Furthermore, it has been reported that the threshold value for allergy induction in humans was about 5000 ppb^41^. Previously, we have found that there is little deposition in organs from the implanted Zr_70_Ni_16_Cu_6_Al_8_ BMG miniscrew, which is not toxic as a metallic biomaterial^15^. In the present study, we measured the elution of metals into blood and compared the blood concentration before and 8 weeks after implantation of miniscrews. Blood concentration of Ni detected was 0.61 ± 0.16 μg/g(610 ppb) with the 1.0 mm diameter Zr_70_Ni_16_Cu_6_Al_8_ BMG miniscrew and 0.72 ± 0.66 μg/g with the 1.3 mm Ti-6Al-4 V miniscrew, which were lower than the normal human blood concentration (1.3–3.3 μg/g, average 2.3 ± 0.16 μg/g)^46^. Blood concentrations of other metals were also at similar levels before and 8 weeks after implantation in Zr_70_Ni_16_Cu_6_Al_8_ BMG and Ti-6Al-4 V miniscrews. These findings suggested that the Zr_70_Ni_16_Cu_6_Al_8_ BMG miniscrew, like the Ti-6Al-4 V miniscrew, is a safe biomaterial for clinical use. Zi-based BMG will make a significant contribution as a biomaterial for orthodontic miniscrew and other medical equipment in the future.

The sample size was determined based on an article that described criteria for biological reproducibility^47^ and other papers performing similar experiments in implant studies with beagle dogs^48,49^. We recognize that the miniscrew sample size in this study is relatively small, however it is considered sufficient to evaluate mechanical properties and biocompatibility of a novel smaller miniscrew made of Zr_70_Ni_16_Cu_6_Al_8_ BMG for orthodontic anchorage, which is the endpoint of the present study. Studies with larger sample sizes are needed to confirm our findings, especially a difference in failure rate, even if the significant results obtained in this study are justified. As the n number of miniscrews increases, the reliability of the significant difference in the results increases. On the other hand, there are practical problems in terms of ethics, costs, and time. In this study, the n number of miniscrews was more than 3 in each group of different type of miniscrews. Therefore, there are the limitations to reliability and reproducibility of data with small numbers of n. The future study will be needed.

In conclusion, Zr_70_Ni_16_Cu_6_Al_8_ BMG had a lower elastic modulus than Ti-6Al-4 V alloy, and the miniscrew made of Zr_70_Ni_16_Cu_6_Al_8_ BMG had sufficient strength for clinical use even with a small diameter of 1.1 mm or less. The Zr_70_Ni_16_Cu_6_Al_8_ BMG miniscrew with a diameter of 1.1 mm or less had higher stability and a lower failure rate than the 1.3 mm diameter Ti-6Al-4 V miniscrew. Furthermore, the Zr_70_Ni_16_Cu_6_Al_8_ BMG miniscrew with a diameter of 1.1 mm or less induced more new bone formation and osseointegration around the miniscrew than the 1.3 mm diameter Ti-6Al-4 V miniscrew. The Zr_70_Ni_16_Cu_6_Al_8_ BMG miniscrew, which has a small diameter and has not been clinically applied to date, has been demonstrated for the first time to be useful for orthodontic anchorage.

## Materials and methods

### Ethics

Animal experiments were performed in accordance with the Regulations for Animal Experiments and Related Activities at Tohoku University. All animal protocols were approved by the Institutional Animal Care and Use Committee of the Tohoku University Environmental and Safety Committee. We complied with the ARRIVE guidelines (Animal Research: Reporting of In Vivo Experiments).

### Preparation of Zr_70_Ni_16_Cu_6_Al_8_ miniscrew

Master alloy ingots of Zr-based metallic glass were prepared by arc-melting a mixture of pure elements in an automatic arc melting furnace with argon atmosphere at the Institute for Materials Research, Tohoku University^15^. Three rods made of five types of Zr-based metallic glass (Zr_55_Ni_5_Cu_30_Al_10_, Zr_60_Ni_10_Cu_20_Al_10_, Zr_65_Ni_10_Cu_17.5_Al_7.5_, Zr_68_Ni_12_Cu_12_Al_8_, and Zr_70_Ni_16_Cu_6_Al_8_) with a diameter of 2.0 mm and a length of 80.0 mm were prepared for measurement of elastic modulus.

Prototype of orthodontic miniscrews made of the Zr_70_Ni_16_Cu_6_Al_8_ BMG with 4 different diameters were prepared by arc-tilt-casting the melt into a copper mold and then machining using a thread rolling method^50,51^. The fabricated Zr_70_Ni_16_Cu_6_Al_8_ BMG miniscrews had a 0.5 mm pitch, and a 5.0 mm in length.

Using Zr_70_Ni_16_Cu_6_Al_8_ BMG, 26 miniscrews with a diameter of 1.3 mm (1.10 mm inner diameter), 28 miniscrews with a diameter of 1.1 mm (0.90 mm inner diameter), 23 miniscrews with a diameter of 1.0 mm (0.8 mm inner diameter), and 27 miniscrews with a diameter of 0.9 mm (0.7 mm inner diameter) were fabricated. Twenty six Ti-6Al-4 V miniscrews (AbsoAnchor II®; Dentos, Daegu, Korea) 1.3 mm in diameter (1.1 mm inner diameters), which are commonly used in clinical practice, were purchased and used for comparison with Zr_70_Ni_16_Cu_6_Al_8_ BMG miniscrews. Zr_70_Ni_16_Cu_6_Al_8_ BMG miniscrews and the Ti-6Al-4 V miniscrew were 0.5 mm in pitch, and 5.0 mm in length.

### Measurement of elastic modulus and torsion test

First, the elastic modulus was measured by a free resonance vibration method (JE-RT; Nihon Techno-Plus Co. Ltd., Osaka, Japan) using three rods made of five types of Zr-based metallic glass.

A torsion test was performed on 0.9 mm, 1.0 mm, and 1.1 mm diameter (0.70 mm, 0.80 mm, 0.90 mm inner diameters, respectively) Zr_70_Ni_16_Cu_6_Al_8_ BMG miniscrews and a 1.3 mm diameter (1.10 mm inner diameter) Ti-6Al-4 V miniscrew using torsion test equipment (PC torque analyser; Vectrix Corporation, Tokyo, Japan) and a digital torque meter (HP-10; HIOS Inc., Tokyo, Japan). The torsion test was performed inserting a dedicated driver tip into the miniscrew head and leaving half of the miniscrew thread out of the jig. Insertion was performed at a speed of 15 rotations/m. A torsional force was applied until the miniscrew broke, and the torque value at the time of breaking was measured. In addition, torsion torque value of miniscrews implanted for 8 weeks into the mandible of a beagle dog was also measured.

### Animals and surgical procedure

Twelve male beagle dogs, 10 months old and weighing 13.0–16.0 kg, were purchased from Kitayama Labs Co Ltd. (Nagano, Japan) and Japan SLC Inc (Shizuoka, Japan), and bred according to the guidelines of Tohoku University animal experiment regulations. The different types of miniscrews used in the experiment were randomly implanted in each side of the mandibular alveolar bone of 10 beagle dogs, and 2 beagle dogs were implanted with either 1.0 mm diameter Zr_70_Ni_16_Cu_6_Al_8_ BMG miniscrews or Ti-6Al-4 V miniscrews for blood samples. Up to 6 miniscrews of different types were randomly implanted in the miniscrew insertion sites in each side of the mandible (Supplementary Fig. S1A).

The experimental groups were 4 groups of Zr_70_Ni_16_Cu_6_Al_8_ BMG miniscrews with diameters of 0.9 mm, 1.0 mm, 1.1 mm, and 1.3 mm; 1 group of 1.3 mm Ti-6Al-4 V miniscrew; a non-loaded group for each miniscrew; and a 200 gf loaded group for each miniscrew, respectively.

All animal experiments were performed under general anesthesia to reduce pain. The dogs were treated with 5 mg/kg Diazepam for preanesthetic medication, 13 mg/kg pentobarbital for anesthesia, and 2 mg/kg xylazine hydrochloride for analgesia. Before inserting miniscrews, dental radiographs of the surgical area were taken to confirm the roots of teeth and the direction of insertion, and the surgical area was sterilized with hydrogen peroxide. After sterilization, local anesthesia was performed with 1.8 ml lidocaine hydrochloride epinephrine/adrenaline injection (Nipro, Osaka, Japan) and an incision was made to the periosteum on the alveolar bone. A pilot hole was drilled into the cortical bone with a low-speed handpiece (500 rpm) and a round bar under water injection. The diameter of the round bar used for the pilot hole was 0.2 mm smaller than the diameter of each miniscrew. A Zr_70_Ni_16_Cu_6_Al_8_ BMG miniscrew or Ti-6Al-4 V miniscrew was inserted in a drilled hole using a manual torque screwdriver (FTD10CN-S; Tohnichi, Tokyo, Japan) at an angle of 45° to the cortical bone surface. To apply a force of 200 gf to the miniscrew, an elastomeric chain (Pro-chain; Dentsply Sirona, Tokyo, Japan) was placed from the miniscrew inserted between the 2nd premolar roots to the miniscrew between the 3rd premolar roots, from the miniscrew inserted between the 3rd and 4th premolars to the miniscrew inserted between the roots of the 4th premolar, and from the miniscrew inserted between the 4th premolar and 1st molar to the miniscrew inserted between the 1st molar roots. The load was measured using a spring scale immediately after implantation; dental radiographs were taken and the root proximity was evaluated. Throughout the experiment, no systemic problems were observed in dogs, such as eating disorders, decreasing body weight or gait disturbance.

### Scanning electron microscopy (SEM) imaging

SEM (JSM-6390LA, JEOL, Tokyo, Japan) was used to evaluate the surface structure of used and unused miniscrews. Used miniscrews harvested 8 weeks after implantation were kept in 70% ethanol at 4 °C until used for SEM imaging. The surface texture of the miniscrew edge, the edge form, and the distance between pitches were examined at low (30×) and high (300×) magnifications.

### Insertion and removal torque testing

The peak value of insertion torque of each miniscrew was measured during miniscrew placement into the alveolar bone using a manual torque screwdriver (FTD10CN-S; Tohnichi, Tokyo, Japan). The removal torque value was also measured at 8 weeks after implantation. Peak insertion and removal torque values were recorded in Ncm.

### Mobility measurement by Periotest

Immediately after the miniscrews were inserted, the mobility of miniscrews was measured at 0 week to evaluate the stability of the miniscrew using the Periotest (Gulden Messtechnik; Bensheim, Germany). The mobility of the miniscrews was also measured at 2, 4, 6 and 8 weeks after implantation. In accordance with the manufacturer's instructions, the tip of the Periotest was applied to the miniscrew head from a distance of 2.0–3.0 mm. Each miniscrew was subjected to Periotest measurements from three directions, approximately 120° apart, on the horizontal plane as reported by Çehreli et al.^52^. The measurement was repeated three times for each direction and average values were determined.

### Evaluation of root proximity and failure rate

The distance between the miniscrew and root surface was calculated using dental radiographs taken after implantation, and defined as (distance between the tip of the miniscrew in the dental radiograph and the surface of the root) x (diameter of the miniscrew)/(diameter of the miniscrew in the dental radiograph).

The dental radiographs were classified into two categories, non-proximity or proximity depending on the distance between the miniscrew tip and root surface (Supplementary Fig. S1B). According to the report of Watanabe et al.^27^, non-proximity was defined as the distance between the miniscrew tip and root surface being more than 0.7 mm, and proximity defined as the distance between the miniscrew tip and root surface being under 0.7 mm (Supplementary Fig. S1B, C). The miniscrew failure rate was calculated and compared with the root proximity and the presence or absence of a 200 gf load.

### Histomorphometric analysis

Eight weeks after implantation, 4.3 mm internal diameter trephine bars (Dentech, Tokyo, Japan) were used for harvesting the alveolar bone specimens including the miniscrew in 12 beagle dogs. A sequence of fluorochrome labels with calcein green (5 mg/kg body weight) (Dojindo Laboratories, Kumamoto, Japan) and tetracycline (10 mg/kg body weight) (Wako, Osaka, Japan) were injected intravenously at 10 days and 3 days, respectively, before harvesting the specimen at 8 weeks post-implantation.

Specimens were immersed in a 4% paraformaldehyde solution for 48 h, dehydrated in an ascending series of ethanol. After dehydration, the specimens were treated with an intermediate agent, xylene, infiltrated with methyl methacrylate (Osteoresin Embedding Kit; Wako, Osaka, Japan) at 4 °C, and embedded at 35 °C. Embedded specimens were sectioned with a Leica Saw Microtome SP1600 (Leica Microsystems, Wetzler, Germany) and stained with Villanueva Osteochrome Bone Stain (Polysciences Inc., Pennsylvania, USA) for bright-field microscopic examination. Thirteen sections with a thickness of 100 μm were prepared every 300 μm from the miniscrew head side of the thread. BIC, BA, MAR and BFR were measured on the 10th, 11th, and 12th sections where the cancellous bone was observed (Supplementary Fig. S2B).

To measure histomorphometric analysis was performed on bone in the 240 μm region from the miniscrew surface using an optical microscope (DP72, Olympus Corporation, Tokyo, Japan) (Supplementary Fig. S2A). Image J Launcher software (National Institutes of Health, Bethesda, Maryland, USA) was used to measure and convert pixels to micrometers. BIC and BA were calculated as averages of 3 sections. Dynamic assessment of bone formation was determined based on dual calcein–tetracycline labeling using a fluorescence microscope (BZ-9000; Keyence, Osaka, Japan). The distance between two consecutive labels was measured for MAR and BFR (Supplementary Fig. S2C, D). MAR was measured with four double labels in the 240 μm region from the miniscrew surface at a magnification of 10 times, and MAR and BFR were calculated as an average value of 12 measured values (4 double labels × 3 sections) (Supplementary Fig. S2C,D).

### Measurement of metal concentration in beagle dogs’ venous blood

Seven Ti-6Al-4 V miniscrews with a diameter of 1.3 mm were implanted in one beagle dog and seven Zr_70_Ni_16_Cu_6_Al_8_ BMG miniscrews with a diameter of 1.0 mm were implanted in another beagle dog. Before implantation and 2, 4, 6, and 8 weeks after implantation, a total of 7.0 ml blood was taken from the saphenous vein of the left hind leg of beagle dogs according to the method of Assad et al.^53^ Blood was taken 4 times and measured. The blood samples were frozen and stored at − 20 °C until analysis. Concentrations of titanium, zirconium, nickel, copper, aluminum, and vanadium within blood were measured by inductively coupled plasma-mass spectroscopy (ICP-MS) (Agilent 8800; Agilent Technologies, California, USA). Analysis by ICP-MS was performed at an infinitesimal material analysis room in the Tohoku University School of Engineering. Metal concentration was measured with blood collected four times from each beagle dog, and the average value and S.D. were calculated. Metal concentrations without any implant were used as controls.

### Statistical analysis

Statistical significance was determined by one-way analysis of variance followed by post hoc analysis using Tukey–Kramer’s multiple comparison tests. The chi-squared or Fisher’s exact probability test were used to examine the correlation between miniscrew failure rate and root proximity. Pearson's correlation coefficient was used to examine the correlation between miniscrew diameter and failure rate, and between miniscrew diameter and root proximity. All data are presented as mean ± standard deviation. Differences were considered statistically significant at *P < 0.05 and **P < 0.01.

## Supplementary Information

Supplementary Information.

## Data Availability

The datasets used and analyzed during the current study available from the corresponding author on reasonable request.

## References

[1] Lesz S, Kwapuliński P (2008). Formation and physical properties of Fe-based bulk metallic glasses with Ni addition. Form. Phys. Prop. Fe-Based Bulk Met. Glas Ni Addit..

[2] Wang YB, Li HF, Cheng Y, Zheng YF, Ruan LQ (2013). In vitro and in vivo studies on Ti-based bulk metallic glass as potential dental implant material. Mater. Sci. Eng. C.

[3] Gebert A (1999). Investigations on the electrochemical behaviour of Zr-based bulk metallic glasses. Mater. Sci. Eng. A.

[4] Kawashima A, Yokoyama Y, Inoue A (2010). Zr-based bulk glassy alloy with improved resistance to stress corrosion cracking in sodium chloride solutions. Corros. Sci..

[5] Yokoyama Y, Yamasaki T, Liaw PK, Inoue A (2008). Study of the structural relaxation-induced embrittlement of hypoeutectic Zr–Cu–Al ternary bulk glassy alloys. Acta Mater..

[6] Yokoyama Y, Fujita K, Yavari AR, Inoue A (2009). Malleable hypoeutectic Zr–Ni–Cu–Al bulk glassy alloys with tensile plastic elongation at room temperature. Philos. Mag. Lett..

[7] Yokoyama Y (2011). Tough hypoeutectic Zr-based bulk metallic glasses. Met. Mater. Trans. Phys. Met. Mater. Sci..

[8] Welsch G, Bunk W (1982). Deformation modes of the α-phase of Ti-6Al-4V as a function of oxygen concentration and aging temperature. Met. Mater Trans. A.

[9] Bowen AW, Stubbington CA (1982). Titanium and Titanium Alloys: Scientific and Technological Aspects.

[10] Yoder GR, Cooley LA, Crooker TW (1978). Fatigue crack propagation resistance of beta-annealed Ti-6Al-4V alloys of differing interstitial oxygen contents. Met. Mater. Trans. A.

[11] Brandes EA (1998). Smithells Metals Reference Book.

[12] Zwicker U (1974). Titan und Titanlegierungen.

[13] Gibson LJ, Ashby MF (1997). Cellular Solids: Structure and Properties.

[14] Lee Y, Welsch G (1990). Young’s modulus and damping of Ti-6Al-4V alloy as a function of heat treatment and oxygen concentration. Mater. Sci. Eng. A.

[15] Ida H (2018). Biosafety, stability, and osteogenic activity of novel implants made of Zr_70_Ni_16_Cu_6_Al_8_ bulk metallic glass for biomedical application. Acta Biomater..

[16] Elias CN, Lima JHC, Valiev R, Meyers MA (2008). Biomedical applications of titanium and its alloys. JOM.

[17] Sundfeldt M, Carlsson LV, Johansson CB, Thomsen P, Gretzer C (2006). Aseptic loosening, not only a question of wear: A review of different theories. Acta Orthop..

[18] Piotrowski B (2014). Interaction of bone–dental implant with new ultra low modulus alloy using a numerical approach. Mater. Sci. Eng. C.

[19] Yamako G (2017). Improving stress shielding following total hip arthroplasty by using a femoral stem made of β type Ti-33.6Nb-4Sn with a Young’s modulus gradation. J. Biomech..

[20] Migliorati M, Drago S, Amorfini L, Nucera R, Silvestrini-Biavati A (2021). Maximum insertion torque loss after miniscrew placement in orthodontic patients: A randomized controlled trial. Orthod. Craniofac. Res..

[21] Chen Y-H (2008). Root contact during insertion of miniscrews for orthodontic anchorage increases the failure rate: An animal study. Clin. Oral Implants Res..

[22] Choa U-H, Yub W, Kyung H-M (2010). Root contact during drilling for microimplant placement. Angle Orthod..

[23] Hwang Y-C, Hwang H-S (2011). Surgical repair of root perforation caused by an orthodontic miniscrew implant. Am. J. Orthod. Dentofac. Orthop..

[24] Suzuki EY, Suzuki B (2011). Placement and removal torque values of orthodontic miniscrew implants. Am. J. Orthod. Dentofac. Orthop..

[25] Jolley TH, Chung CH (2007). Peak torque values at fracture of orthodontic miniscrews. J. Clin. Orthod..

[26] Kuroda S (2007). Root proximity is a major factor for screw failure in orthodontic anchorage. Am. J. Orthod. Dentofac. Orthop..

[27] Watanabe H (2013). Orthodontic miniscrew failure rate and root proximity, insertion angle, bone contact length, and bone density. Orthod. Craniofac. Res..

[28] Santiago RC, de Paula FO, Fraga MR, Picorelli Assis NMS, Vitral RWF (2009). Correlation between miniscrew stability and bone mineral density in orthodontic patients. Am. J. Orthod. Dentofac. Orthop..

[29] Suzuki M (2013). Evaluation of optimal length and insertion torque for miniscrews. Am. J. Orthod. Dentofac. Orthop..

[30] Park H-S, Jeong S-H, Kwon O-W (2006). Factors affecting the clinical success of screw implants used as orthodontic anchorage. Am. J. Orthod. Dentofac. Orthop..

[31] Büchter A (2005). Load-related implant reaction of mini-implants used for orthodontic anchorage. Clin. Oral Implants Res..

[32] Wilmes B, Panayotidis A, Drescher D (2011). Fracture resistance of orthodontic mini-implants: A biomechanical in vitro study. Eur. J. Orthod..

[33] Muguruma T (2011). Relationship between the metallurgical structure of experimental titanium miniscrew implants and their torsional properties. Eur. J. Orthod..

[34] Iijima M (2008). Torsional properties and microstructures of miniscrew implants. Am. J. Orthod. Dentofac. Orthop..

[35] Motoyoshi M, Hirabayashi M, Uemura M, Shimizu N (2006). Recommended placement torque when tightening an orthodontic mini-implant. Clin. Oral Implants Res..

[36] Meredith J (1998). Building operations management theory through case and field research. JOM.

[37] Favero LG, Pisoni A, Paganelli C (2007). Removal torque of osseointegrated mini-implants: An in vivo evaluation. Eur. J. Orthod..

[38] Ivanoff C-J, Sennerby L, Johansson C, Rangert B, Lekholm U (1997). Influence of implant diameters on the integration of screw implants: An experimental study in rabbits. Int. J. Oral Maxillofac. Surg..

[39] Olivé J, Aparicio C (1990). Periotest method as a measure of osseointegrated oral implant stability. Int. J. Oral Maxillofac. Implants.

[40] Harmankaya N, Igawa K, Stenlund P, Palmquist A, Tengvall P (2012). Healing of complement activating Ti implants compared with non-activating Ti in rat tibia. Acta Biomater..

[41] Basketter DA, Briatico-Vangosa G, Kaestner W, Lally C, Bontinck WJ (1993). Nickel, cobalt and chromium in consumer products: A role in allergic contact dermatitis?. Contact Derm..

[42] Gawkrodger DJ (1993). Nickel sensitivity and the implantation of orthopaedic prostheses. Contact Derm..

[43] Haudrechy P, Foussereau J, Mantout B, Baroux B (1994). Nickel release from nickel-plated metals and stainless steels. Contact Derm..

[44] Kanerva L (1994). Nickel release from metals, and a case of allergic contact dermatitis from stainless steel. Contact Derm..

[45] Kinbara M (2011). Allergy-inducing nickel concentration is lowered by lipopolysaccharide at both the sensitization and elicitation steps in a murine model. Br. J. Dermatol..

[46] Minoia C (1990). Trace element reference values in tissues from inhabitants of the European community: I—A study of 46 elements in urine, blood and serum of Italian subjects. Sci. Total Environ..

[47] Naegle K, Gough NR, Yaffe MB (2015). Criteria for biological reproducibility: What does ‘n’ mean?. Sci. Signal..

[48] Deguchi T (2003). The use of small titanium screws for orthodontic anchorage. J. Dent Res..

[49] Deguchi T (2008). Histomorphometric evaluation of alveolar bone turnover between the maxilla and the mandible during experimental tooth movement in dogs. Am. J. Orthod. Dentofac. Orthop..

[50] Yokoyama Y, Inoue K, Fukaura K (2002). Pseudo float melting state in ladle arc-melt-type furnace for preparing crystalline inclusion-free bulk amorphous alloy. Mater. Trans..

[51] Pater Z, Gontarz A, Weroñski W (2004). New method of thread rolling. J. Mater. Process Technol..

[52] Çehreli S, Arman-Özçırpıcı A (2012). Primary stability and histomorphometric bone-implant contact of self-drilling and self-tapping orthodontic microimplants. Am. J. Orthod. Dentofac. Orthop.

[53] Assad M (2003). Porous titanium-nickel for intervertebral fusion in a sheep model: Part 2: Surface analysis and nickel release assessment. J. Biomed. Mater. Res. Part B Appl. Biomater..

