# Supplementary material for: Mechanical properties and biocompatibility of a novel miniscrew made of Zr70Ni16Cu6Al8 bulk metallic glass for orthodontic anchorage
Source: Sci Rep. 2023 Feb 21;13:3038. doi: 10.1038/s41598-023-30102-3 (PMC9943840; doi:10.1038/s41598-023-30102-3)

**Supplementary Figure legends:**

**Supplementary Figure 1. Schematic diagrams and dental radiographs of the implantation site and proximity to the root**

A: A schematic diagram of the miniscrew implantation site. The implantation is indicated by a red circle between the 2nd premolar roots, the 3rd premolar roots, the 3rd premolar and the 4th premolar roots, the 4th premolar roots, the 4th premolar and the 1st molar roots, and the 1st molar roots. Different types of miniscrews were implanted randomly in the alveolar bone. 1.3 mm diameter of Ti-6Al-4V miniscrews and 1.3 mm, 1.1 mm, 1.0 mm, and 0.9 mm diameter Zr_70_Ni_16_Cu_6_Al_8_ BMG miniscrews were pre-drilled with round bars having diameters of 1.1 mm, 1.1 mm, 0.9 mm, 0.8 mm, and 0.7 mm, respectively. B: Schematic diagrams showing the positional relationship between the miniscrews and the tooth roots. Left: non-proximity, the distance from the miniscrew tip to the root surface was 0.7 mm or more; Right: proximity, the distance from the miniscrew tip to the root surface was less than 0.7 mm. C: Dental X-ray photograph showing the positional relationship between the miniscrews and the tooth roots. Left: non-proximity, Right: proximity. A total of 90 miniscrews were used to evaluate root proximity and miniscrew failure rates. Scale bar, 5.0 mm

**Supplementary Figure 2. Bone histomorphometric analysis region around the miniscrew**

A: Arrowhead indicates new bone formation site with purple dyeing. BIC, BA, MAR, and BFR were measured in the region within 240 μm of the miniscrew surface. B: Thirteen sections with a thickness of 100 μm were prepared every 300 μm from the miniscrew head side of the thread. Tenth, 11th, and 12th sections from the head side of the thread were used for the measurement of BIC, BA, MAR, and BFR. C: MAR was measured with four double labels in the 240 μm region divided 4 at a magnification of 10 times, and MAR was calculated as an average value of 12 measured values (4 double labels × 3 sections). Double label distance, single label distance, and bone surface was measured at a magnification of 10 times, and BFR was calculated (BFR μm/day; MAR × (double label surface + 1/2 single label surface) / bone surface × 100). D: The distance between labels of calcein green (a) and tetracycline yellow (b) was measured for MAR. M, Miniscrew; AB, Alveolar bone (orange or dark green stained area); BM, Bone marrow, Scale bars: A, C: 250 μm; D: 100 μm

**Supplementary Figures:**

Supplementary Figure. 1


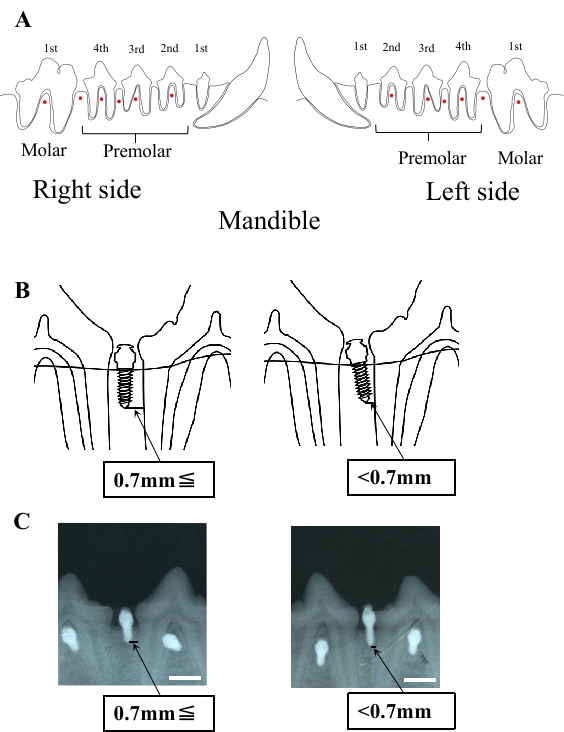


**Supplementary Figure. 2**


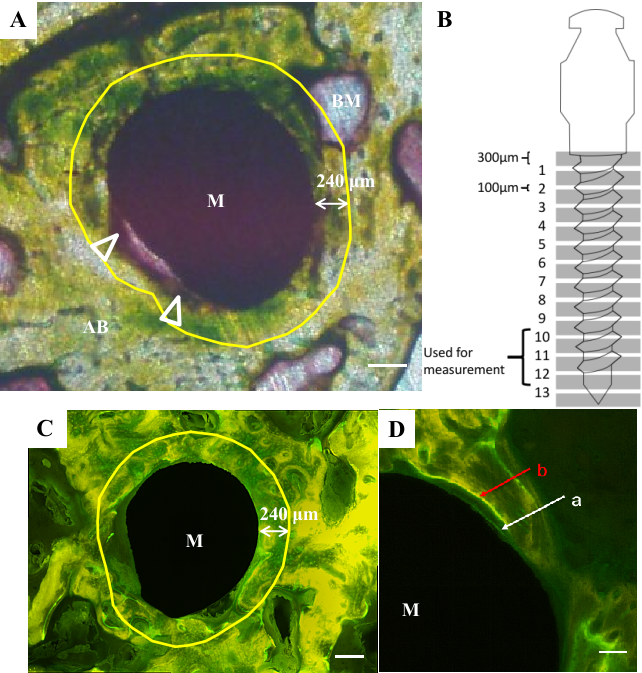

Supplement: Supplementary file 1 — Supplementary Information. [file 41598_2023_30102_MOESM1_ESM.docx]
